# Objective Assessment of shoulder mobility with a new 3D gyroscope - a validation study

**DOI:** 10.1186/1471-2474-12-168

**Published:** 2011-07-21

**Authors:** Bilal Farouk El-Zayat, Turgay Efe, Annett Heidrich, Udo Wolf, Nina Timmesfeld, Thomas J Heyse, Stefan Lakemeier, Susanne Fuchs-Winkelmann, Markus D Schofer

**Affiliations:** 1 Department of Orthopaedics and Rheumatology, University Hospital Marburg, Baldingerstrasse, 35033 Marburg, Germany; 2 Institute for Medical Biometry and Epidemiology, Philipps University Marburg, Bunsenstraße 3, 35037 Marburg, Germany; 3 Department of Physiotherapy, University Hospital Marburg, Baldingerstrasse, 35033 Marburg, Germany

## Abstract

**Background:**

Assessment of shoulder mobility is essential for clinical follow-up of shoulder treatment. Only a few high sophisticated instruments for objective measurements of shoulder mobility are available. The interobserver dependency of conventional goniometer measurements is high. In the 1990s an isokinetic measuring system of BIODEX Inc. was introduced, which is a very complex but valid instrument. Since 2008 a new user-friendly system called DynaPort MiniMod TriGyro ShoulderTest-System (DP) is available. Aim of this study is the validation of this measuring instrument using the BIODEX-System.

**Methods:**

The BIODEX is a computerized robotic dynamometer used for isokinetic testing and training of athletes. Because of its size the system needs to be installed in a separated room. The DP is a small, light-weighted three-dimensional gyroscope that is fixed on the distal upper patient arm, recording abduction, flexion and rotation. For direct comparison we fixed the DP on the lever arm of the BIODEX. The accuracy of measurement was determined at different positions, angles and distances from the centre of rotation (COR) as well as different velocities in a radius between 0° - 180° in steps of 20°. All measurements were repeated 10 times. As satisfactory accuracy a difference between both systems below 5° was defined. The statistical analysis was performed with a linear regression model.

**Results:**

The evaluation shows very high accuracy of measurements. The maximum average deviation is below 2.1°. For a small range of motion the DP is slightly underestimating comparing the BIODEX, whereas for higher angles increasing positive differences are observed.

The distance to the COR as well as the position of the DP on the lever arm have no significant influence. Concerning different motion speeds significant but not relevant influence is detected. Unfortunately device related effects are observed, leading to differences between repeated measurements with any two different devices up to 8° at maximal range of motion (180°).

**Conclusions:**

In summary the results shows high correlation and good reproducibility of measurements. All deviations are inside the tolerance interval of 5°, if one device is used. An unlikely systematic device effect is detected. These laboratory trials are promising for the validation of this system in humans. The challenge for both systems will be the changing of the COR in the shoulder joint at elevations higher than 90°.

## Background

Rehabilitation has had a continuing interest in the measuring of outcomes especially because of competitive different therapies and necessity of cost-effectiveness [1]. The current consensus states that functional activity is the most important outcome to measure improvement of rehabilitation [2,3].

The shoulder joint allows as a combination of five different joints and sliding surfaces the highest range of motion (ROM) in the human body. The univocal terminology of joint motion introduced by the American Academy of Orthopaedic Surgeons in 1965 to standardize terminology for two-dimensional (2D) movement has found widespread clinical acceptance, as they need description during physical examination. However, when monitoring shoulder movements of daily life which typically are three-dimensional (3D) [4], the subjective assessment of axial rotation about the humerus' long axis is rather vague [5]. Hence objective assessment of shoulder joint mobility especially after conservative or operative therapy is very demanding. Unfortunately the correct assessment of shoulder mobility is crucial for evaluation of approved shoulder scores (e.g. Constant-Score, Rowe-Score or Simple Shoulder test).

Existing objective physical rehabilitation outcome instruments in the daily practice are time consuming, complicated, expensive and not applicable [6,7]. Human motion analysis systems need cable wires, synchronization, external references, mounting sensors to the subject etc.. All these make the use of motion analysis unnecessarily difficult. Moreover manual goniometers can measure joint angles only statically and have a low interindividual reliability and reproducibility [8]. This bias is in shoulder patients with decreased mobility even higher [9].

Since the late 1960s objective instruments were designed mostly for isokinetic testing and training [10] in exercise sciences. Several studies tried to find instruments for a convergent, objective measure on the amount of extremity use, especially in neurorehabilitation and orthopaedic follow up [7]. An external infrared marker system with video monitoring and computer evaluation was introduced in 1990 for kinematic assessment of lower extremity joint angle motion and implemented later on also for upper extremities [11].

A new small and handy 3D accelerometer called DynaPort MiniMod TriGyro ShoulderTest of McRoberts Inc., The Hague, Netherlands (DP) [12] was designed to assess upper extremity function and was introduced recently. Aim of this study is to define the accuracy and validity of this instrument [13] at laboratory conditions that never has been done before.

## Methods

### Devices

#### BIODEX

Isokinetic dynamometers provide constant velocity with accommodating resistance throughout a joint's ROM. This resistance is provided using an electric or hydraulic servo-controlled mechanism at a user-defined constant velocity. This type of muscle contraction has become a popular method to assess dynamic muscle function and joint movement in both clinical and research settings. With the interfacing of isokinetic dynamometers and microprocessors, objective measurements of human muscle function and ROM can be obtained.

The BIODEX 3 System (Biodex) isokinetic dynamometer (Biodex Medical Systems, Shirley, New York, USA) is an actual update of the first multi-mode computerized robotic dynamometer worldwide. It consists of a special seat on which the patient is fixed with belts, a lever arm that could be positioned at different joints at different angles and a computer unit with special software. The total operating floor space required for this system is 64 sq ft (6 sq m). It has an electrically controlled servomechanism and could be used with different modes for different modalities and phases of rehabilitation. The isokinetic resistance mode provides impact-free acceleration and deceleration. The reactive eccentric mode is for submaximal neuromuscular re-education in the early phases of rehabilitation and the passive motion mode with very slow speeds is ideal for proprioceptive testing and training. Further on there is an isometric mode commonly used pre- and postoperatively and an isotonic mode. An optional software allows researchers to customize motor control, movement tracking and data analysis.

The test person is positioned on the seat and fixed with belts to avoid evasive movement of the trunk. The lever arm is adapted to the extremity length and the centre of rotation (COR) of the concerned joint is defined. For tests of the upper extremity the handle allows free rotation for pro- and supination in the elbow and wrist joint. The results are directly visualised on the systems screen and could be printed.

In several studies the Biodex has been shown to be a reliable and valid instrument for the measurement of human joint function [14,15].

### DynaPort MiniMod TriGyro ShoulderTest

**Figure 1:**
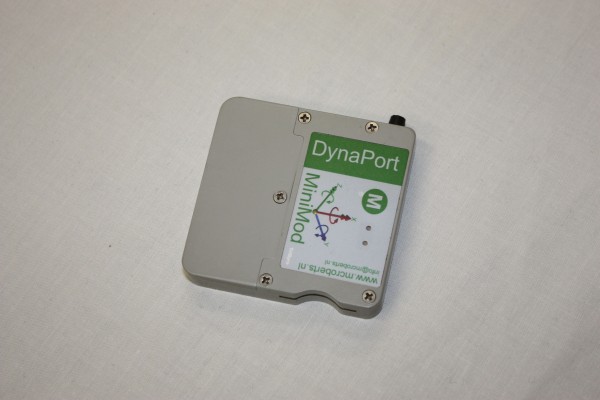
**Three-dimensional Gyroscope DynaPort MiniMod TriGyro ShoulderTest**.

The DP is a small box (62 × 41 × 18 mm, 53 grams) containing three gyroscopic sensors (Figure 1). A gyroscope is a device for measuring or maintaining orientation, based on the principles of conservation of an angular momentum. A mechanical gyroscope is essentially a spinning wheel or disk whose axle is free to take any orientation. This orientation changes much less in response to a given external torque than it would without the large angular momentum associated with the gyroscope's high rate of spin. Since external torque is minimized, its orientation remains nearly fixed, regardless of any motion of the platform on which it is mounted. Traditional applications of gyroscopes include ship navigation or stabilization of flying vehicles.

The three DP gyroscopes can measure all rotation and angular velocity which can be converted then to angle information by a special mathematical algorithm. The only preparation needed to calculate angles is to teach the device the axis of the shoulder joint by performing a calibration procedure in two directions (e.g. elevation and abduction).

The DP is fixed to the distal upper arm with a flexible belt. Subsequently the calibration procedure is executed by consecutively movement of the arm in one plane up to an angle of 90° (abduction and flexion). The proper assessment is than performed with five repetitions in each direction. After using matrix algebra and goniometric operations the movement is expressed in the elevation and simultaneous internal- and external rotation of the upper arm as a mean value of these five repetitions. The suppression of measurement error is done using single value decomposition.

Because of small size of the device and battery operation, assessment is possible in every location for up to 72 hours continuously. The raw data is stored on a commercially available secure digital (SD) card. Using special software (MiRA^®^, McRoberts Inc., The Hague, Netherlands) the measurement calibration is checked and could be adapted. In a second step all results could be displayed and evaluated. Another possibility is to perform a digital encryption of the data and upload at the company's homepage for analysis. Subsequent a PDF-file with relevant processed data is sent back within few minutes via e-mail.

### Data acquisition - Set up

For direct comparison of both devices we fixed the DP at the rectangular lever arm of the Biodex and added a professional water level as a conventional goniometer for additional randomized monitoring. For differentiation whether the position of the DP on the lever arm or the distance to the COR of the Biodex affects the DP measurement, we repeated all tests at four different positions on the rectangular lever arm (medial, lateral, anterior and posterior), as well as at different distances from the COR (12 and 24 cm). For evaluating the influence of motion velocity on the accuracy of the measurements we performed all assessments at three different speeds (30°/s, 45°/s and 60°/s) of the Biodex. For exact evaluation of the accuracy at different movement angles, all assessments were made in steps of 20° between 0° and 180° and additionally at 90°. The definition of the 90° position was suspected to be crucial for changing of the COR in the human shoulder joint at this position.

After performing a power analysis for defining the number of repetitions needed, each measurement was performed ten times at every position (medial, lateral, anterior and posterior) and distance (12 and 24 cm from COR) as well as velocity (30°/s, 45°/s and 60°/s) and angle (0°-180°); that resulted in a total of more than 4000 measurements. The tolerated accuracy was defined as below 5° deviation between both systems.

**Figure 2:**
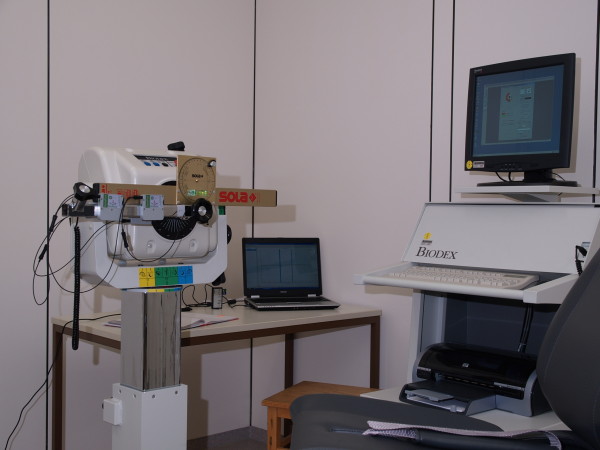
**Laboratory setting with BIODEX, DP, water level and computer**.

For gaining time, four DP-devices were fixed at different positions and distances from the COR at the lever arm at once (Figure 2).

### Calibration

To start an assessment series calibration of both systems is necessary. The Biodex needs a definition of start and end position (e.g. 0° and 180°), which is manually entered. Before starting measurements at Biodex the force (moment) exerted on the dynamometer arm and recorded by the sensors as well as the angular position and velocity of the lever arm are adjusted daily.

The DP calibration is performed standardized by a single "flexion" and "abduction" movement to adjust the gyroscopes in three dimensions. After fixing the DP-System at Biodex-lever arm the SD memory-card is inserted. By that the system is initializing. 30 seconds later two markers are placed to the DP by pushing the button. The lever arm is moved into flexion up to 45° and back to starting point. Another marker is placed and the lever arm is moved into abduction. After getting back to neutral position a third marker is placed and the DP is ready for assessments in flexion and extension axis. For calibration of abduction movements the direction on the lever arm has to be changed to be rectangular to the flexion axis. After fixing the DP at the lever arm calibration in the abduction axis is performed likewise.

The calibration procedure is highly sensitive for disturbances and noise in the signal, why it is very important to perform movements only in one axis. Further on the manufacturer recommends performing all calibration movements higher than 40° for better accuracy.

### Assessments

The measurement starts by automatic movement of the Biodex lever arm at default speed up to the prior defined position (angle). The DP measurement consists of five repetitions at same velocity and same ROM. Out of these five values a mean is calculated. This protocol was repeated ten times for each position, each velocity and each angle with a new calibration prior to every series. The duration to execute the protocol for one parameter (one position, one velocity, one distance and one angle) is about 10 minutes.

### Statistical analysis

At different settings, which are defined as a combination of position, velocity and distance from COR, measurements at angles between 20° and 180° in steps of 20° and at 90° were performed. As no significant differences in the assessments of abduction and flexion were found, both were combined for the following analyses. This resulted in a performance of all trials at 24 different settings (4 positions, 3 velocities and 2 distances from COR) at every measured angle (ten different angles).

To evaluate the accuracy of DP a linear regression model of DP on Biodex was performed. For each of the total of 24 settings separate intercepts and slopes of the curve were estimated. Since earlier graphical evaluation suggested device specific effects, random effects for devices and positions, as well as for devices, positions and angles on Biodex were included in this model. The statistical expression for that is:

To investigate the influence of each parameter (position, distance, velocity and angle) on slope and intercept likelihood ratio tests were done. Reproducibility was described by prediction intervals for the difference between two measurements by changing just one parameter.

## Results

### Calibration

**Figure 3:**
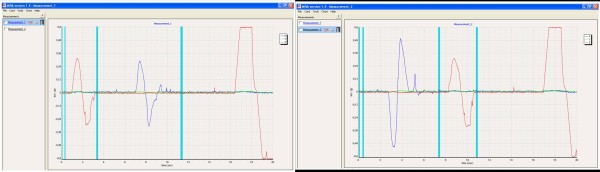
**Calibration-check by MiRA^®^-software: left for flexion and right for abduction; the turquoise vertical lines show the placed markers**.

The quality of calibration is evaluated by special software (MiRA^®^, Figure 3). The registered course of movement is visualized in two-dimensional graphs. The placed markers (vertical bars) are important to define start and end of measurements as well as changes in movement direction.

**Figure 4:**
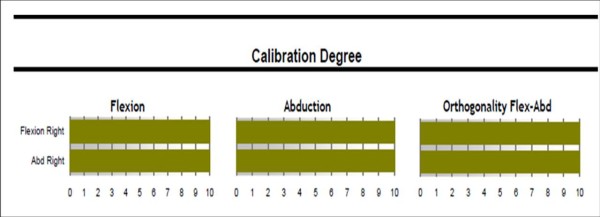
**Calibration quality numeric scale (provided in company's sent back-report)**.

Another option to evaluate the quality of calibration is set on the reports sent back by the company's data sheet (Figure 4). The quality of calibration based on the amount of unwished rotation is presented in a numeric scale. In an optimal case the calibration movement is performed without any internal or external rotation. The "Orthogonality Flex-Abd" bar shows highest results, if flexion and abduction movements are performed at right angles to each other.

The results of the calibration procedure in the presented assessments were in the mean satisfactory at 9.8 (Range: 8.0 - 10.0). If the calibration of a measurement was below 8 the whole assessment was repeated.

### Accuracy

For describing accuracy of DP slopes and intercepts of the fitted linear model for each setting were considered. The optimal condition showing no difference between DP and Biodex would result in a slope of 1 and an intercept of 0. Table 1 demonstrates all slopes and intercepts for each setting.

**Table 1:** Slopes and intercepts for 24 settings at different parameters (velocity, distance from COR, position)

position of DP	distance from COR [cm]	velocity [°/s]	slope	intercept
		30	1.03	-1.03
		
	**12**	45	1.03	-1.04
		
**anterior**		60	1.02	-1.54
	
		30	1.03	-0.94
		
	**24**	45	1.03	-0.99
		
		60	1.03	-1.31

		30	1.02	-1.38
		
	**12**	45	1.01	-1.39
		
**medial**		60	1.01	-1.59
	
		30	1.02	-1.29
		
	**24**	45	1.02	-1.26
		
		60	1.02	-1.66

	**12**	30	1.02	-1.20
		
		45	1.02	-1.27
		
**posterior**		60	1.02	-1.71
	
	**24**	30	1.02	-1.20
		
		45	1.02	-1.33
		
		60	1.02	-1.56

	**12**	30	1.02	-0.96
		
		45	1.02	-0.97
		
**lateral**		60	1.02	-1.46
	
	**24**	30	1.02	-1.27
		
		45	1.02	-1.25
		
		60	1.02	-1.69

**Figure 5:**
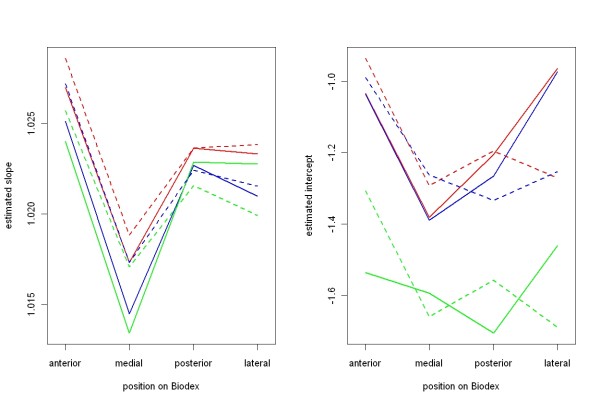
**Graphical illustration of slopes and intercepts for 24 settings with different parameters (velocity, distance from COR, position): red = 30°/s, blue = 45 °/s, green = 60°/s, dotted lines = 24 cm distance from COR, solid lines = 12 cm distance from COR**.

The data of table 1 is graphically visualized in Figure 5. Here is obviously seen that the medial position seem to be the best, since the slope for all velocities and distances from COR at this position is nearest 1. Even in other positions of the DP on the lever arm of the Biodex the maximal slope is not higher than 1.03. No significant differences between the *four positions *for slopes (P = 0.09) and intercepts (P = 0.09) could be found.

For *different distances from COR *no significances were found for slopes (P = 0.12) and intercepts (P = 0.09).

Evaluation of *different velocities *showed significant differences in slopes (P < 0.0001) and intercepts (P < 0.0001). Calculation of estimated mean values was performed exemplarily for medial position and showed mean differences between DP and Biodex up to 2.1° (Table 2.).

**Table 2:** Effect of different velocities and distances from COR at medial position of DP on accuracy; predicted mean values on DP from linear regression model

		estimated angle on DP at velocity of		
**Distance from COR**	**Angle on Biodex**	**30°/s**	**45°/s**	**60°/s**

	20°	18.97°	18.90°	18.68°
	
12 cm	90°	90.18°	89.92°	89.62°
	
	180°	181.74°	181.22°	180.82°

	20°	19.08°	19.08°	18.68°
	
24 cm	90°	90.40°	90.30°	89.88°
	
	180°	182.10°	181.86°	181.42°

### Reproducibility

Concerning reproducibility of DP measurements the influence of velocity and between different devices was assessed, because both other parameters (position and distance from COR) can be fixed in a setting with subjects. For quantification of the influence of velocity, 95%-prediction intervals for differences between repeated measurements at different velocities (30°/s vs. 60°/s) and constant other parameters (medial position, 12 cm distance from COR and same device) were calculated. The results are presented exemplarily for three relevant angles (20°, 90° and 180°) showing differences up to 2.3° as maximum value (Table 3).

**Table 3:** 95%-prediction intervals for influence of velocity and device effects ^a^- when same device is used for both measurements at 12 cm distance from COR ^b^- when same velocity and distance from COR is used for both measurements

	95%-prediction intervals for differences at medial position	
**angle on Biodex**	**between 30°/s and 60°/s^a^**	**between two devices^b^**

**20°**	-1.04° - 1.62°	-2.11° - 2.11°

**90°**	-0.77° - 1.90°	-4.33° - 4.33°

**180°**	-0.42° - 2.25°	-8.02° - 8.02°

Concerning investigations on a possible device-related effect 95%-prediction intervals for differences between repeated measurements with different devices were calculated keeping all other parameters constant. The results show, that at higher angles intervals getting wider with upper limits from 2.11° up to 8.02° (Table 3).

## Discussion

Objective assessment of shoulder motion is crucial for diagnostics, follow-up, quality-control and rehabilitation. Most of existing objective instruments are time consuming, complicated, expensive and not applicable in daily practice [6,7]. The "gold standard" method with manual goniometers has a low interindividual reliability and reproducibility [8,9].

Advances in engineering and computer sciences have made systems more versatile and much more accurate. However, most of the systems are no more purely measuring one specific variable, but provide several data more. At the same time the systems are getting smaller and more user-friendly what have made them easier for day to day use.

Aim of this study is the validation of the small and handy DP at abduction and flexion movements to a reference (Biodex) at laboratory conditions.

### Calibration

Calibration movements (e.g. flexion/abduction) should be performed in a rectangle for getting highest accuracy. As manufacturer's recommendations on calibration procedure suggests to perform all movements higher than 40° for ensuring accuracy, a negative effect in calibrations below that angle was not found. Moreover it is recommended, that the velocity of calibration movement should not be too slow, without defining it. The presented data shows no relevant influence of velocity on accuracy of calibration.

In comparing the calibration quality over time of assessments, a steep learning curve was observed, what could be remarked in lesser repetitions of calibration with ongoing measures.

It should be mentioned, that in regular settings the calibration procedure cannot be checked by the observer. It is intended by the manufacturer that all measured data are uploaded to a central internet server and sent back after analysis via e-mail. Insufficient calibration can be remarked first when assessments and data upload are completed. In that case complete assessment series have to be repeated. In the presented setting, the analysing software was provided for direct analysis in laboratory, but was still first viewable after end of examination. An optical feedback control on the device showing immediately the quality of calibration (good, modest, bad) could be a practical solution.

### Accuracy

There are several potential sources of error that can threaten the credibility of assessments. Machine linked inconsistencies like differences between tests on the same device during normal operation or during calibration procedure. These could relate to both, the tested (DP) and the testing device (Biodex). That's why the Biodex system was maintained and adjusted by a company technician just prior to this study for ensuring accuracy. Different studies proved high reliability and validity of Biodex [14,16]. Sinacore et al. recommended that calibration should be carried out on the moment measuring model every testing day and the velocity of the arm, every test velocity [17]. The needed calibration before starting measurements at Biodex was performed every testing day and for velocity prior to every test.

On the other hand subject variations in humans (motivation, pain, fatigue, etc.) when performing repeated or multiple measurements as well as testing procedure errors (poor/inconsistent stabilization) as possible sources of error were excluded from the study design at laboratory conditions.

Further sources of error could be observer dependent or related to data processing (e.g. software), different fixation of DP-devices at lever arm, different velocities of movement as well as different distances from COR, or even an effect of the device (DP) itself. Intratester bias [18] was excluded as the same observer performed all measurements. The analysing software has not changed over study time. A possible software error would result in a constant bias, which cannot be excluded in this study.

An important finding is that all slopes are greater than 1 but very close to 1. Optimal slope would be exactly 1; worst slope found in this study is at anterior position with a value of 1.03. Best slope is found at medial position (1.01) what suggests this position as best. All intercepts between different positions are smaller than 0 but are differing in a minimal extend between 1.0° and 1.7°. Differences in intercepts are in comparing issues not relevant, for cancellation of intercepts in subsequent assessments.

Observed slopes and intercepts for all settings show that for small ROM DP is slightly underestimating "real" values of the Biodex and vice versa overestimating higher ROMs. Different positions of DP on the lever arm of the Biodex, as well as different distances from COR showed no significant influence on slope and intercept of estimated regression lines. That means, that due to gyroscope rotation in all three planes the positioning of the DP and its orientation in space seems to be correct. Only significant influence for both slope and intercept were found for different velocities.

The overall differences at different velocities and distances from COR are very small, ranging at medial position of DP in the mean from 1.3° underestimation up to 2.1° overestimation. Concerning relevance of slope and intercept at repetitive measurements describing e.g. a treatment course, the amount of in- or decrease of mobility is interesting. This would result in a cancellation of intercepts in subsequent assessments. That means that the slope is the most important factor in means of accuracy. There is no good explanation for the systematic bias of under- and overestimation (in minimal extend) at different angles. The findings suggest that the error could be related to the length of the trajectory of the movement. This may be due to the mathematical algorithm of data processing, which should be re-evaluated for possible optimization.

Noticeable is that medial and lateral as well as anterior and posterior position have similar results for estimated slopes. This seems to be logical as these positions are in opposite to each other. There is no good explanation for differences of positions of DP on the lever arm, as gyroscopes should retain their angular momentum and keep orientation, regardless of any motion of the platform on which they are mounted.

The manufacturer recommends fixing the device at the lateral lower upper arm. That would correspond to lateral position in the presented settings. The results do not show a clear superiority of this position, which has to measure at least equal values as the perfectly opposite medial position. In practical use a medial placement of the device on the lower upper arm will not be applicable and will disturb measurements as the arm will be in slight abduction at starting position. Therefore a lateral position, as recommended, should be preferred, at the expense of little lower accuracy.

### Reproducibility

Standardisation of device position and distance from COR at laboratory conditions, as well as on subjects is easy possible. The reproducibility of assessments within an institution is only dependent on movement velocity, if always same device is used. For comparison of assessments between institutions a device related effect must be added.

Relevant device-related effects were detected. If two assessments with any 2 devices are performed to a maximal ROM of 180°, differences between both devices reaches up to 8°, which is ahead of the prior defined tolerance interval of 5°.

If subsequent assessments were performed at different velocities with the same device, only a difference of up to 2.2° is recognized.

This means that repeated assessments in one institution should be performed with the same device to get comparable results. For practical clinical applications this would be tolerable.

Unfortunately multi-centre trials or comparison of assessments between centres will not be as accurate as necessary.

## Conclusion

In summary the new and handy DP is a very interesting device with easy application and high user friendliness. The results of this study show that DP represents the ROM of the reference movements with reasonable accuracy. Especially due to simple handling and short duration of testing this method is promising to be applicable in clinical routine. The definite benefit of the DP is the easy and inexpensive application, what makes it affordable even for physiotherapists and physician offices for objective evaluation of shoulder mobility during therapy. Up to now this was only possible in huge scientific institutions, because of high investment and demanding handling.

The results of this study can only be generalized to the mechanical measurement capabilities of this DP gyroscope and accompanying software, which could be optimized by the manufacturer to eliminate the existing discrepancies. The accuracy is compared to the huge Biodex system reasonable. It is very hopeful that the presented results seem to be very systematic which makes it eventually possible to correct them by improving the device itself or the mathematical algorithm, by which the raw data is calculated. A challenging point will be the detection and treatment of the device related effects.

Future studies must incorporate human participants to determine the reliability and validity of this instrument at assessing clinically relevant measurements of human shoulder joint function with special attention placed on abduction higher than 90°. This position will state a challenge at comparing both systems in humans for changing of the COR of the shoulder joint at that angle. Other possibilities could include optic and video monitoring systems [19] from kinematical studies in comparison to the DP. Moreover the practicability in day to day use must be clarified.

Future implementations of DP could include real-time applications or feedback to the subject wearing it (balance control, limitation of ROM in shoulder rehabilitation, etc.).

## Competing interests

The authors declare that they have no competing interests.

## Authors' contributions

All authors read and approved the final manuscript. BFE participated in the design of the study, carried out the study, interpretation of the results and drafted the manuscript. TE helped with the draft of the manuscript and interpretation of the results. AH carried out the study and participated in interpretation of the results as well as in the draft of the manuscript. UW participated in the design of the study and operation of the Biodex. NT set up the protocol and performed the statistical data analysis. TJH and SL helped with the statistical data analysis and the draft of the manuscript. SFW participated in the design of the study and interpretation of the results. MDS participated in the design of the study, carried out the study, interpretation of the results and helped with the draft of the manuscript.

## Pre-publication history

The pre-publication history for this paper can be accessed here:

http://www.biomedcentral.com/1471-2474/12/168/prepub
